# Multidrug-Resistant IncA/C Plasmid in *Vibrio cholerae* from Haiti

**DOI:** 10.3201/eid2011.140889

**Published:** 2014-11

**Authors:** Jason P. Folster, Lee Katz, Andre McCullough, Michele B. Parsons, Kristen Knipe, Scott A. Sammons, Jacques Boncy, Cheryl Lea Tarr, Jean M. Whichard

**Affiliations:** Centers for Disease Control and Prevention, Atlanta, Georgia, USA (J.P. Folster, L. Katz, M.B. Parsons, K. Knipe, S.A. Sammons, C.L. Tarr, J.M. Whichard);; International Health Resources Consulting, Atlanta (A. McCullough);; Laboratoire National de Santé Publique, Port-au-Prince, Haiti (J. Boncy)

**Keywords:** Vibrio cholerae, antimicrobial resistance, multidrug-resistant plasmid, bacteria, Haiti, IncA/C plasmid

**To the Editor:** The agents of epidemic cholera are *Vibrio cholerae* toxigenic serogroups O1 and O139. Cholera symptoms include watery diarrhea and severe dehydration, which can rapidly result in death unless rehydration therapy is prompt (*1*). Antimicrobial agents may reduce the severity and duration of disease (*1*); commonly used are tetracyclines, fluoroquinolones, macrolides, and trimethoprim/sulfamethoxazole (*1*). However, *V. cholerae* resistance to antimicrobial drugs is increasing because of the accumulation of genetic mutations and the acquisition of resistance genes, which are usually transferred on mobile genetic elements such as integrating conjugative elements (ICEs) (*1*).

As of March 12, 2014, the ongoing cholera outbreak that began in Haiti in October 2010 had caused 700,796 cases and 8,548 deaths (*2*). To characterize infections, the National Public Health Laboratory in Haiti and the US Centers for Disease Control and Prevention (CDC) collaborated to perform standard microbiological and antimicrobial-drug susceptibility testing on isolates from case-patients. Since October 2010, the National Public Health Laboratory has identified 465 isolates, which were then forwarded to CDC for determination of MICs for 15 antimicrobial agents by broth microdilution (Sensititer; Trek Diagnostics Systems, Cleveland, OH, USA) according to manufacturer’s recommendations (Table). Resistance was defined by the Clinical and Laboratory Standards Institute interpretive standards, when available (*3*). The typical outbreak strain (2010EL-1786) displayed resistance to streptomycin, sulfisoxazole, trimethoprim/sulfamethoxazole, and nalidixic acid, and decreased susceptibility to ciprofloxacin and chloramphenicol (*4*). Resistance was caused by mutations in the QRDR regions of the *gyrA* and *parC* genes and presence of ICE*Vch*Hai1 containing the *dfrA1*, *floR*, *strAB*, and *sul2* resistance genes (*4*).

**Table:** Susceptibility, resistance genes, and plasmids associated with Haiti *Vibrio cholerae* outbreak isolates

Isolate	Resistance profiles [MIC mg/L]*	Resistance genes/mutations	Plasmids
*V. cholerae* 2010EL-1786	STR [>64], FIS [>256], TMP/SXT [>4], CIP(I) [0.5], NAL [>32]	*strAB sul2, dfrA1, floR, gyrA*(S83I)/*parC*(S85L)	None
*V. cholerae* 2012EL-2176	AMP [>32], AMC [>32], CRO [>64], FOX [32], TIO [>8], CHL(I) [16], GEN [>8], STR [>64], FIS [>256], TMP/SXT [>4], TET(I) [8], CIP(I) [0.5], NAL [>32]	*bla_CMY-2_, bla_CTX-M-2_, bla_TEM-1_, floR, aac*(*3)-IIa ,strAB, sul2, dfrA1, dfrA27, tetA, mphA, gyrA*(S83I)/*parC*(S85L)	IncA/C2
*Escherichia coli* DH10B p2012EL-2176	AMP [>32], AMC [>32], CRO [>64], FOX [>32], TIO [>8], AZM [>16], CHL [>32], GEN [>8], STR [>64],† FIS [>256], TMP/SXT [>4], TET [32]	*bla_CMY-2_, bla_CTX-M-2_, bla_TEM-1_, mphA, floR, aac(3)-IIa. strAB, sul2, dfrA27, tetA*	IncA/C2

*Drugs tested were AMP, ampicillin; AMC, amoxicillin/clavulanic acid; AZM, azithromycin; CHL, chloramphenicol; CIP, ciprofloxacin; CRO, ceftriaxone; FIS, sulfisoxazole; FOX, cefoxitin; GEN, gentamicin; KAN, kanamycin; NAL, nalidixic acid; STR, streptomycin; TET, tetracycline; TIO, ceftiofur; and TMP/SXT, trimethoprim/sulfamethoxazole; S83I, serine-to-isoleucine change at amino acid position 83; S85L, serine-to-leucine change at amino acid position 85. Drugs that yielded intermediate results are followed by (I). Clinical Laboratory Standards Institute break points are not established for AZM. The AZM break points used in this study (>16 mg/L) were established by the National Antimicrobial Resistance Monitoring System and should not be used to predict clinical efficacy.  †*E. coli* DH10B is intrinsically resistant to STR.

In April of 2012, the 2 agencies began sentinel laboratory-based surveillance for acute diarrheal disease at 4 hospitals in Haiti (*5*). As part of this surveillance, fecal specimens were sent to the National Public Health Laboratory for organism isolation, identification, antimicrobial-drug testing, and subsequently to CDC for expanded antimicrobial-drug testing and molecular characterization. One isolate, 2012EL-2176, showed the typical resistance phenotype of the outbreak strain but additional resistance to ampicillin, amoxicillin/clavulanic acid, cefoxitin, ceftriaxone, ceftiofur; the tetracycline MIC was intermediate (Table).

Analysis of this isolate by serotype, pulsed-field gel electrophoresis, multilocus variable number–tandem repeat analysis, and whole-genome sequencing confirmed that the isolate was similar to outbreak isolates (data not shown) (*6*). PCR and whole-genome sequencing analysis by use of ResFinder (http://www.genomicepidemiology.org/) identified the original outbreak resistance determinants and additional determinants (*aac(**3**)-IIa*, *bla*_CMY-2_, *bla*_CTX-M-2_, *bla_TEM-1_, dfrA15*, *mphA*, *sul1, and tetA*) (*7*). Plasmid transfer by electroporation into *Escherichia coli* (DH10B) confirmed that the resistance determinants were plasmid encoded. PCR-based replicon testing identified an IncA/C2 plasmid, and PCR and whole-genome sequencing confirmed that the plasmid encoded a unique set of resistance determinants (*aac(**3**)-IIa*, *bla*_CMY-2_, *bla*_CTX-M-2_, *bla*_TEM-1_, *dfrA15*, *mphA*, *sul1*, and *tetA*) and a second copy of the resistance genes *floR*, *strAB*, and *sul2* identical to those located in ICE*Vch*Hai1 (*8*). Antimicrobial-drug susceptibility testing of the transformant demonstrated transfer of the resistance profile and additional resistance to chloramphenicol, tetracycline, and decreased susceptibility to azithromycin (Table). The lack of association between the presence of the resistance determinants *floR* and *tetA* and the lack of resistance in *V. cholerae* has been observed previously, possibly because of lower gene expression (*9*). The plasmid was mobilizable by conjugation (conjugation efficiency = 1.3–1.4 × 10^−2^) when *E. coli* J53 was used as the recipient.

IncA/C plasmids are widespread in *Enterobacteriaceae* and commonly confer multidrug resistance. BLASTn (http://blast.ncbi.nlm.nih.gov/Blast.cgi) comparison of the completed plasmid p2012EL-2176 sequence with the National Center for Biotechnology Information nucleotide collection showed similarities to other IncA/C-*bla*_CMY_ plasmids; most similarity (total score = 3.009 × 10^5^) was to pAR060302, found in an *E. coli* isolate from a dairy calf (*10*). Most IncA/C-*bla*_CMY_ plasmids have 3 resistance regions: *sul2* region (*floR*-*tetA*-*strAB*-*sul2*), *cmy-2* insertion region, and Tn21-like region (*aad*-*aac*). Plasmid p2012EL-2176 contains the *sul2* and *cmy-2* insertion regions and a putative *arr3*-*drfA27*-*aadA16*-*sul1* resistance gene cassette at the Tn-21 location. This plasmid has additional resistance gene insertions: a putative cassette containing a *bla_TEM-1_* and *aac(**3**)-IIa* gene upstream of the *sul2* region and insertions of *bla_CTX-M-2_*, *sul1*, and *mphA* genes downstream of the *arr3*-*drfA27*-*aadA16*-*sul1* cassette (Technical Appendix).

Since discovery of isolate 2012EL-2176, sentinel surveillance has not detected increased antimicrobial-drug resistance among *V. cholerae* in Haiti. The ability of IncA/C plasmids to acquire novel resistance cassettes from multiple sources makes it difficult to hypothesize as to the origins of plasmid p2012EL-2176. Although this plasmid was most closely related to a plasmid found in *E. coli*, it was also closely related to plasmids in *Salmonella*, *Klebsiella*, and *Providencia*. *Enterobacteriaceae* are found in the environment and/or in the host gut; therefore, the isolate could have acquired the plasmid in the environment or within the host. The latter scenario would limit the possible spread of this plasmid and could explain its rarity. The original Haiti outbreak isolate has been shown to be poorly naturally transformable, accounting for the lack of acquired chromosomal genes and nearly homologous genomic content among outbreak isolates (*6*). Therefore, the acquisition of plasmids, and their resistance genes, may represent the major source of future variability among *V. cholerae* involved in the Haiti outbreak.

Technical Appendix Additional methods and comparison of plasmid p2012EL-2176 and plasmid pAR060302.
