# Supplementary material for: Multidrug-Resistant IncA/C Plasmid in Vibrio cholerae from Haiti
Source: Emerg Infect Dis. 2014 Nov;20(11):1951–3. doi: 10.3201/eid2011.140889 (PMC4214316; doi:10.3201/eid2011.140889)
Supplement: Technical Appendix — Additional methods and comparison of plasmid p2012EL-2176 and plasmid pAR060302. [file 14-0889-Techapp-s1.pdf]

Article DOI: http://dx.doi.org/10.3201/eid2011.140889

Multidrug-Resistant IncA/C Plasmid in *Vibrio cholerae* from Haiti

Technical Appendix

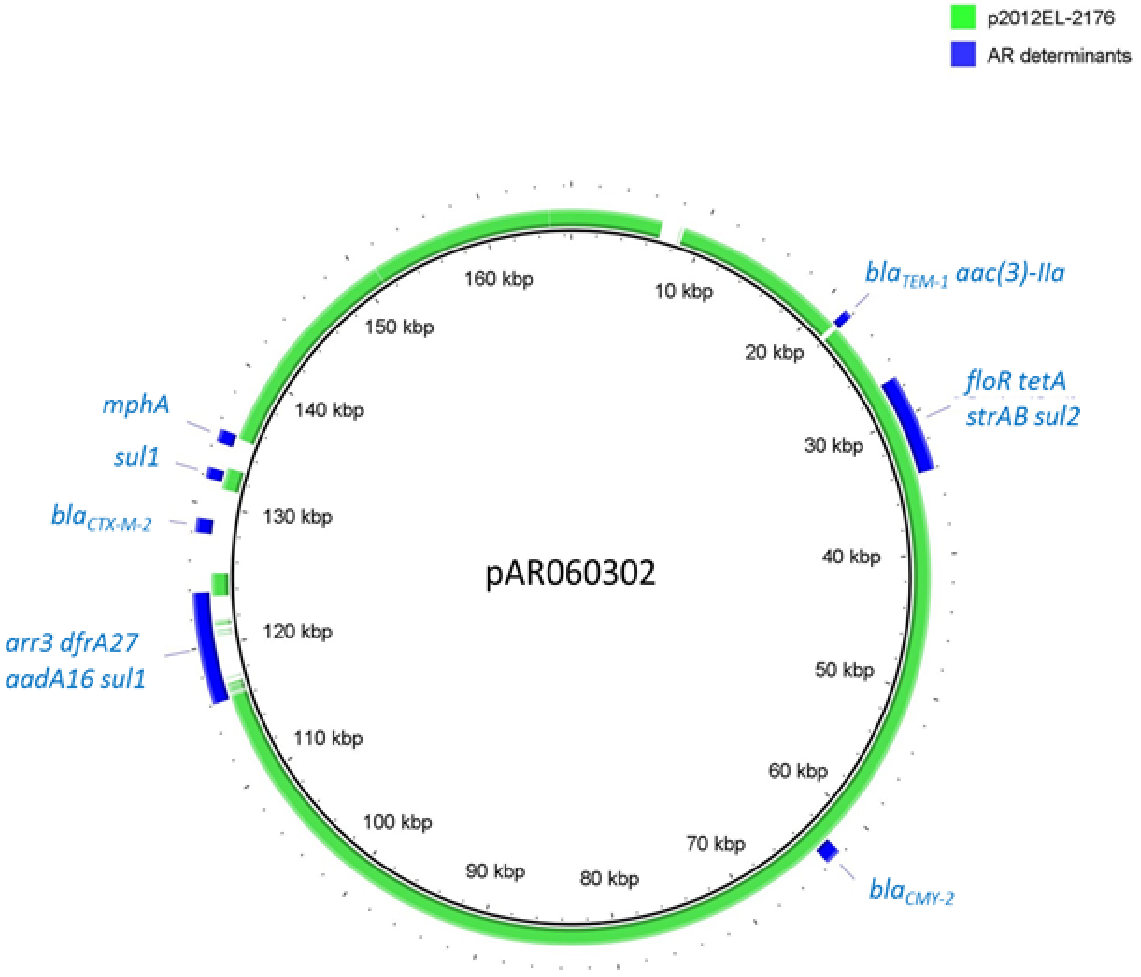

Technical Appendix Figure. Comparative analysis of plasmid p2012EL-2176 to plasmid pAR060302. The genome was sequenced by using NexteraXT library kits, paired-end, 150-bp reads using a MiSeq (Illumina, San Diego, CA, USA) (GenBank accession nos. CP007634 and CP007635) and on 4 SMRT cells on the Pacific Biosciences *RS* (Pacific Biosciences, Menlo Park, CA, USA) and assembled with the HGAP1 protocol (P_PreAssembler for error correction, Celera Assembler for assembly of corrected reads). A 35× long-read cutoff (8,855 bp) was used in the P_PreAssembler, and the longest (15.**3**×) of the corrected reads were assembled by Celera. The assembly yielded 1 plasmid contig (GenBank accession

no. CP007636). A BLAST comparison was performed by using BLASTN with a cutoff value of 70% identity and pAR060302 as the reference sequence. The green circle shows the regions of p2012EL-2176 with identity to pAR060302. The blue inserts show regions of p2012EL-2176 containing antimicrobial-drug resistance (AR) genes. The circular plot was generated by using BLAST Ring Image Generator (BRIG) software (*1*).

Additional methods:

The conjugation experiment used *V. cholerae* 2012EL-2176 as the donor and *E. coli* J53 (sodium azide R) as the recipient (*2*).

The 3 resistance regions on IncA/C-cmy plasmids are described in this publication (*3*).

References

1. Alikhan NF, Petty NK, Ben Zakour NL, Beatson SA. BLAST Ring Image Generator (BRIG): simple prokaryote genome comparisons. BMC Genomics. 2011.12:402. http://dx.doi.or/10.1186/1471-2164-12-402

2. Martínez-Martínez L, Pascual A, Jacoby GA. Quinolone resistance from a transferable plasmid. Lancet. 1998;351:797–9. PubMed http://dx.doi.org/10.1016/S0140-6736(97)07322-4

3. Johnson TJ, Lang KS. IncA/C plasmids: An emerging threat to human and animal health? Mob Genet Elements. 2012;2:55–8. PubMed http://dx.doi.org/10.4161/mge.19626
